# Hypertension and Heart Failure: From Pathophysiology to Treatment

**DOI:** 10.3390/ijms25126661

**Published:** 2024-06-17

**Authors:** Giovanna Gallo, Carmine Savoia

**Affiliations:** Clinical and Molecular Medicine Department, Faculty of Medicine and Psychology, Sant’Andrea Hospital, Sapienza University of Rome, 00189 Rome, Italy; giovanna.gallo@uniroma1.it

**Keywords:** hypertension, heart failure, HFpEF, HFrEF, antihypertensive treatment

## Abstract

Hypertension represents one of the primary and most common risk factors leading to the development of heart failure (HF) across the entire spectrum of left ventricular ejection fraction. A large body of evidence has demonstrated that adequate blood pressure (BP) control can reduce cardiovascular events, including the development of HF. Although the pathophysiological and epidemiological role of hypertension in the development of HF is well and largely known, some critical issues still deserve to be clarified, including BP targets, particularly in HF patients. Indeed, the management of hypertension in HF relies on the extrapolation of findings from high-risk hypertensive patients in the general population and not from specifically designed studies in HF populations. In patients with hypertension and HF with reduced ejection fraction (HFrEF), it is recommended to combine drugs with documented outcome benefits and BP-lowering effects. In patients with HF with preserved EF (HFpEF), a therapeutic strategy with all major antihypertensive drug classes is recommended. Besides commonly used antihypertensive drugs, different evidence suggests that other drugs recommended in HF for the beneficial effect on cardiovascular outcomes exert advantageous blood pressure-lowering actions. In this regard, type 2 sodium glucose transporter inhibitors (SGLT2i) have been shown to induce BP-lowering actions that favorably affect cardiac afterload, ventricular arterial coupling, cardiac efficiency, and cardiac reverse remodeling. More recently, it has been demonstrated that finerenone, a non-steroidal mineralocorticoid receptor antagonist, reduces new-onset HF and improves other HF outcomes in patients with chronic kidney disease and type 2 diabetes, irrespective of a history of HF. Other proposed agents, such as endothelin receptor antagonists, have provided contrasting results in the management of hypertension and HF. A novel, promising strategy could be represented by small interfering RNA, whose actions are under investigation in ongoing clinical trials.

## 1. Introduction

Heart failure (HF) is a complex and rapidly increasing syndrome, with an estimated prevalence of 1.1–5.5%, accounting for 30 to 64 million people worldwide [1].

Hypertension represents one of the main and most common risk factors leading to the development of HF across the entire spectrum of left ventricular ejection fraction (LVEF), but with a fundamental role in HF with preserved EF (HFpEF, LVEF ≥ 50%) [2,3].

The age-standardized prevalence rate of hypertensive heart disease has been estimated at 217.9 per 100,000 people, with the individual and global disease burden increasing with patient and population aging [4].

Among the people from the Framingham Heart Study cohort, 91% of HF patients had a history of hypertension in the previous 20 years [5,6]. Moreover, epidemiological studies have shown that the risk of developing HF was increased by twofold in hypertensive men and by threefold in women, with hypertension accounting for 39% of HF cases in men and 59% in women [7]. Consistently, the prevention and accurate treatment of hypertension and other risk factors, such as diabetes and obesity, was associated with a 35-year HF-free survival (34.7 and 38.0 years in men and women, respectively) in subjects aged 45 years with an 86% lower risk or incident HF across the remaining life course [8].

The pathophysiological and epidemiological role of hypertension in the development of HF is well known. It is primarily related to chronic left ventricular (LV) pressure overload and increased intravascular volume, affecting the ventricular structure and function through different molecular mechanisms [6]. In hypertensive patients who develop LV hypertrophy (LVH) the risk of death or non-fatal complications is increased by two- to four-fold independently of age, gender and other risk factors [9]. In addition, hypertensive acute HF is characterized by a high rate of rehospitalization (22% to 30% in the first three months) and by a high rate of both in-hospital (from 3.8% to 11%) and one-year (from 20% to 36%) mortality [10].

Undoubtedly, blood pressure (BP) control with the available antihypertensive drugs is crucial to prevent cardiovascular events and HF, as suggested by different clinical trials and by the recent guidelines [11,12]. Moreover, BP control obtained with antihypertensive drugs or drugs with BP-lowering properties commonly used in HF, such as type 2 sodium glucose transporter inhibitors (SGLT2i), is also important in patients with overt HF to reduce cardiovascular mortality [13,14].

Nevertheless, some critical issues still deserve clarification, including BP targets in HF patients at increased risk of poor outcomes [10].

In this review article, we will discuss the role of hypertension in developing heart failure phenotypes, and the available evidence about the favorable effects of BP control with antihypertensive drugs in preventing HF and cardiovascular events. Furthermore, we will discuss the recent evidence on the effectiveness of BP-lowering drugs in hypertensive heart disease and overt HF, the role of new promising pharmacological strategies in HF, and the recommended BP target levels in patients with HF.

## 2. Hypertension and Heart Failure Phenotypes

Hypertension-related cardiac remodeling is a dynamic process, and phenotypic transitions have been described. Pressure overload mainly leads to concentric LVH, characterized by increased cardiac mass at the expense of chamber volume. This condition leads to diastolic dysfunction, which is the consequence of an impaired distensibility of the LV, with reduced relaxation and increased filling pressure independently of the contractile function of the LV and its symptoms [15]. This condition eventually leads to HFpEF, which is characterized by increased LV mass with about-normal LV systolic function and symptoms of HF. HFpEF may evolve to HF with reduced ejection fraction (HFrEF) with the progression of the disease under certain circumstances, including poor BP control and comorbidities [15]. This condition is characterized by reduced LV systolic function, eccentric hypertrophy, and LV dilatation (increased cardiac mass and chamber volume) [16]. Additional risk factors, such as older age, ethnicity, overweight or obesity, and concomitant diseases, such as diabetes, coronary artery disease, and chronic kidney disease, contribute to increased hemodynamic stress and the transition to different phenotypes of hypertensive cardiomyopathies and HF [15,17]. Messerli and colleagues proposed four stages of hypertension-related HF: stage I: consisting of isolated LV diastolic dysfunction without LVH; stage II: characterized by LV diastolic dysfunction with concentric LVH; stage III: in which clinical HF (dyspnea and pulmonary edema) is associated with concentric LVH; stage IV: with the presence of eccentric LVH and HF with reduced EF (HFrEF, LVEF < 40%) [6].

LVH is a consequence of an increased cardiac myocyte size and myocardial fibrosis associated with augmented extracellular connective tissue and matrix metalloproteinase-2 (MMP-2) activity, activation of the phosphoinositide 3-kinase (PI3K)/protein kinase B (AKT), and protein kinase C (PKC) pathways, abnormal Ca2+ homeostasis, mitochondrial dysfunction, oxidative stress, and apoptosis [18,19,20,21,22]. In such a context, neurohormonal dysfunction with overactivation of the renin-angiotensin-aldosterone system (RAAS) and the sympathetic nervous system (SNS) plays a pivotal role in growth and hypertrophic signaling. In particular, angiotensin II, through its G-protein-coupled type 1 receptor, activates extracellular signal-regulated kinase (ERK)/mitogen-activated protein kinases (MAPKs), peroxisome proliferator-activated receptor-γ coactivator-1α and 1β (PGC-1α and PGC-1β), and mechanistic target of rapamycin (mTOR) pathways, leading to the stabilization of the IкB kinase (IKK) complex, the activation of the nuclear factor kappa-light-chain enhancer of activated B cells (NF-κB) and calcineurin and calmodulin kinase II (CaMKII), and finally, to hypertrophic gene expression [23,24,25,26,27].

LV diastolic dysfunction with LVH is likely the key feature of HFpEF. Several other structural and functional abnormalities have been described, including subtle LV systolic dysfunction, left atrial (LA) impairment, abnormal right ventricular pulmonary artery coupling, pulmonary vascular disease, systemic vascular stiffening, and coronary and peripheral microvascular dysfunction [17].

**Figure 1:**
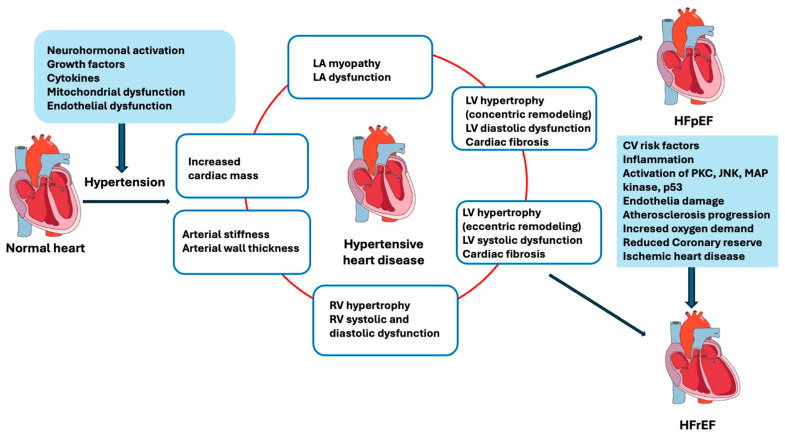
Progression from hypertension to heart failure. CV; cardiovascular; HFpEF; heart failure with preserved ejection fraction; HFrEF; heart failure with reduced ejection fraction; JNK, c-Jun N-terminal kinase; LA, left atrium; LV, left ventricle; MAP, mitogen-activated protein kinase; PKC; protein kinase C; RV, right ventricle.

Besides LVH and diastolic dysfunction, other pathophysiological processes are involved in the development of HF, such as increased vascular stiffness, systemic microvascular endothelial inflammation, and capillary rarefaction leading to oxygen delivery impairment and afterload increase [16,17] (Figure 1).

Pulmonary hypertension, secondary to increased LV and LA pressures, may also lead, in some circumstances, to pulmonary vascular disease, which is common in HFpEF, accounting for up to 80% of patients. These clinical conditions may play a pivotal role in the clinical scenario with impaired recruitment of LV preload due to excessive right heart congestion and blunted right ventricular systolic reserve [28].

Despite a normal LVEF, alterations in stress-corrected endocardial and mid-wall shortening, twisting, or circumferential and longitudinal shortening (by using tissue Doppler or strain imaging) have been described in HFpEF, contributing to impaired cardiac output, elevation in LV filling pressures, and decreased exercise capacity [29,30,31]. Abnormal peripheral vasorelaxation increased LV afterload and impaired LV contractile reserve contribute to the limitation of stroke volume reserve during exertion and to systolic dysfunction in HFpEF. Increased vascular stiffness raises LV afterload and LV systolic elastance with an unfavorable role in LV early relaxation and contractile function [32,33,34]. Moreover, coronary microvascular inflammation and decreased nitric oxide (NO) bioavailability cause coronary microvascular dysfunction and rarefaction, contributing to cardiac injury and to a mismatch between increased myocardial oxygen demand and decreased coronary reserve, which may exacerbate LV systolic dysfunction [35,36,37].

The persistence of increased afterload, associated with neurohormonal dysregulation, and water and salt retention lead to the progression of cardiac injury with loss of myocyte cells, fibrosis, change in ventricular shape with LV dilatation, reduced efficiency of myocardial contraction, and finally, to HFrEF [6]. At the molecular level, mitochondrial dysfunction and type 1 angiotensin II receptor downstream effectors increase reactive oxygen species (ROS) production and nuclear levels of oxidatively modified guanine products with ROS-induced DNA and RNA damage. Oxidative damage contributes to impairment of the electron transport chain, to mitochondrial uncoupling, and finally to bioenergetic dysfunction, which promotes cell death. [38,39]. In addition, shear stress due to hypertension is associated with the activation of different biological pathways, such as PKC epsilon, c-Jun N-terminal kinase (JNK), MAP kinase, and p53, contributing to atherosclerotic plaque progression and a further worsening of endothelial function [40,41,42], contributing to the progression to HFrEF (Figure 1).

## 3. Blood Pressure Treatment and HF

A large body of evidence has demonstrated that adequate BP control can reduce cardiovascular events, including the development of HF. A meta-analysis including 613,815 participants has shown that every 10 mm Hg reduction in systolic BP (SBP) significantly reduced the risk of HF by 28% [43]. Therefore, according to European guidelines, BP lowering should be prioritized over selecting specific antihypertensive drug classes because treatment benefit largely originates from BP reduction [16].

Five major drug classes, including angiotensin-converting enzyme inhibitors (ACEi), angiotensin receptor blockers (ARB), beta-blockers, calcium channel blockers (CCB), and thiazide/thiazide-like diuretics, have effectively reduced BP and cardiovascular events in randomized clinical trials [11]. Hence, these drugs and their combinations are recommended as the basis of antihypertensive treatment strategies [11]. The initiation of therapy with a two-drug combination is recommended for most hypertensive patients, and the preferred associations should comprise an RAAS blocker (either an ACEi or an ARB) with a CCB or thiazide/thiazide-like diuretic [11]. Beta-blockers should be used at the initiation of therapy or any treatment step in HFrEF and as anti-ischemic treatment [11].

If BP is not controlled with the initial two-drug combination by using the maximum recommended and tolerated dose of the respective components, treatment should be increased to a three-drug combination. Single-pill combinations should be preferred at any treatment step to promote therapeutic adherence [11]. Although there is no specific indication to prefer one antihypertensive class over another, a network meta-analysis of 223,313 patients showed that diuretics, ACEi, and ARBs represented the most efficient classes of drugs to reduce HF onset compared with placebo (odds ratio [OR], 0.59, 0.71, and 0.76, respectively) as well as being more effective than calcium channel blockers in preventing HF development [44].

Guidelines also recommend lifestyle interventions combined with pharmacological treatment to reduce BP. These measures include weight reduction in overweight or obese subjects, healthy dietary patterns preferring vegetables, fruits, vegetable oils, fish, and poultry among meat products, salt intake reduction, and daily physical activity of at least moderate intensity [11].

### 3.1. BP Target Levels and HF Prevention

Some controversy exists regarding the BP targets to prevent cardiovascular outcomes in hypertensive patients, particularly for HF development. Different randomized interventional trials have investigated the impact of BP-lowering treatments on HF. The Cardio-Sis (Studio Italiano Sugli Effetti CARDIOvascolari del Controllo Della Pressione Arteriosa SIStolica trial) study, which enrolled 1111 nondiabetic hypertensive patients, evaluated the effects of an SBP reduction < 130 mm Hg compared to usual BP control with a target < 140 mm Hg on HF hospitalizations [45]. Patients randomized to tight BP control experienced a reduced incidence of the study outcomes without reaching statistical significance. In the ACCORD BP (Action to Control Cardiovascular Risk in Diabetes Blood Pressure) study, including diabetic patients at high cardiovascular risk, intensive BP lowering treatment with an SBP target < 120 mm Hg had neutral effects on HF prevention compared to standard therapy [46]. In the CHARM-preserved program (Candesartan in HF: Assessment of Reduction in Mortality and morbidity study), a BP target < 130/80 mm Hg in the candesartan subgroup significantly prevented hospital admissions for chronic HF [47]. The Aldo-DHF (Aldosterone Receptor Blockade in Diastolic Heart Failure) study also showed that lowering BP < 130/80 mm Hg might improve LV diastolic function [48]. Therefore, although the available evidence is not unequivocal, a tight blood pressure control seems to be suggestable for HF prevention and HF hospitalizations reduction. In this regard, the SPRINT (Systolic Blood Pressure Intervention Trial) study conducted in nondiabetic subjects found that an intensive antihypertensive strategy (SBP target < 120 mm Hg) significantly reduced the primary composite endpoint of myocardial infarction, other acute coronary syndromes, stroke, HF, or cardiac death compared to standard treatment [49,50]. Also, regarding HF as a separate endpoint, a strict BP control showed greater efficacy than a non-intensive BP strategy. A subgroup analysis of the study showed that intensive BP treatment could significantly regress LVH and improve cardiac function, further preventing symptomatic HF. In addition, an intensive BP lowering strategy also reduced the risk of acute HF hospitalizations in both HFpEF and HFrEF among older, nondiabetic patients at increased risk of cardiovascular disease [51].

Consistently, the STEP (Strategy of Blood Pressure Intervention in Elderly Hypertensive Patients) study, which enrolled elderly patients aged >60 years, demonstrated a reduced incidence of HF in the group randomized to a BP target < 130 mm Hg [52]. Thus, tight BP control seems to protect more against the development of HF in patients with a large spectrum of risk factors, even in relatively old patients.

Therefore, based on these results, European and US guidelines recommend a BP target less than 130/80 mm Hg with a word of caution regarding BP below 120/70 mm Hg for hypertensive patients at high risk of HF [11,12]. Caution should be used in older patients in whom the primary blood pressure recommended goal is <140/80 mm Hg, but a target < 130/80 mm Hg can be considered if treatment is well tolerated. Nevertheless, in frail patients, blood pressure targets should be individualized (Table 1). In this regard, little and not conclusive evidence exists on the BP targets in special populations, including older hypertensive patients or patients with different degree of frailty [51,53]. In very frail patients, treatment targets should be individualized, starting antihypertensive drugs with lower doses and up-titrating treatment more slowly [11].

### 3.2. BP Management in HF Patients

It should be underlined that the management of hypertension in HF relies on the extrapolation of findings from high-risk hypertensive patients in the general population and not from specifically designed studies in HF populations [53].

In HFrEF and HFpEF settings, the BP threshold and target for treatment do not differ from those recommended for general cardiovascular prevention by antihypertensive treatment if well tolerated [11,12]. However, observational studies have described a J-shaped relationship between SBP and all-cause and cardiovascular mortality in patients with HF, particularly for HFrEF [54]. Nevertheless, whether low BP values might have an adverse prognostic significance in HFpEF remains an unsolved question. In such a context, it has been hypothesized that low SBP might represent a more advanced disease condition, with a poorer prognosis being a surrogate marker for more severe heart dysfunction and lower cardiac output in HFrEF [55]. It should be noted that, in case of non-severe and asymptomatic hypotension while taking drugs recommended for HFrEF treatment, it is suggested to maintain the same drug dosage. In case of symptomatic or severe persistent hypotension (SBP  < 90 mm Hg), decreasing dosage or dismissing BP lowering drugs not indicated for HFrEF treatment is recommended. The dosage of loop diuretic should also be titrated in the absence of associated signs of congestion [56].

The choice of the best therapeutic strategies to prevent the development of LVH and HFpEF in hypertensive patients and to control BP once HFpEF has already occurred represents an issue of particular interest. Pharmacological and non-pharmacological treatments for HF aim to increase survival, reduce hospitalizations for worsening HF, and improve quality of life. In such a context, different pharmacological strategies with BP-lowering effects are approved by the international EU for the management of HF with reduced EF (LVEF < 40%), including beta-blockers, angiotensin receptor-neprilysin inhibitors (ARNi, sacubitril/valsartan), mineralocorticoid receptor antagonists (MRA), and SGLT2i [13,14]. However, besides SGLT2i, no significant benefits have been demonstrated with the other abovementioned drugs in the management of HFpEF. On the other hand, a recent meta-analysis including 30,882 HFpEF patients showed that treatment with neurohormonal inhibitors (RAAS blockers and ARNi) was significantly associated with a reduced risk of death and HF hospitalizations [57]. Table 2 summarizes old and new BP-lowering drugs with current or potential use in hypertension and HF.

In patients with hypertension and HFrEF, European guidelines recommend combining drugs with documented outcome benefits, including ACEi (ARBs if not tolerated), substituted eventually by ARNi, which has shown efficacious antihypertensive effects [13,14]. However, whether this agent could prevent HF in hypertensive patients is uncertain, and further clinical trials are needed. Beta-blockers, MRA, and SGLT2i, if not contraindicated and well tolerated [11,13], are also strongly recommended.

If adequate BP control is not achieved despite the up-titration of agents of these five major disease-modifying drug classes along with the use of additional treatment with a diuretic to manage fluid balance, a dihydropyridine CCB can be added in HFrEF [11].

In patients with HFpEF, a therapeutic strategy with all major antihypertensive drug classes (ACEi or ARBs, beta-blockers, CCBs, and thiazide/thiazide-like diuretics) is recommended [11]. In addition, SGLT2i are recommended independently from the presence of diabetes. The substitution of ACEi/ARBs by ARNi and treatment with an MRA independent of resistant hypertension can be considered, particularly in the lower HFpEF spectrum [11].

ARNi have been demonstrated to exert greater BP-lowering effects compared to RAAS inhibitors due to neprilysin inhibition. Indeed, neprilysin inhibition increases biologically active natriuretic peptides, inducing diuresis, natriuresis, and vasodilation, decreasing vascular stiffness and oxidative stress, and counterbalancing RAAS, the sympathetic nervous system (SNS), endothelin, and vasopressin [58]. In a population of 1328 patients with mild and moderate hypertension, Ruilope and colleagues showed a more significant BP reduction in the sacubitril/valsartan group compared to valsartan [59]. Also, the phase II PARAMOUNT (Prospective comparison of ARNI with ARB on Management Of heart failUre with preserved ejection fracTion) trial, which randomized 301 participants, demonstrated that after 12 weeks of treatment, SBP reduction was significantly greater in the sacubitril/valsartan group compared with valsartan (29.3 vs. 22.9 mm Hg, respectively), these results persisting at 36 weeks [60]. In a post hoc analysis of the PARAGON-HF (Prospective Comparison of ARNI With ARB Global Outcomes in Heart Failure With Preserved Ejection Fraction) trial, the proportion of HFpEF patients with resistant hypertension who achieved an effective BP control was greater in the sacubitril/valsartan group compared to valsartan [61].

These results support the use of sacubitril/valsartan as BP-lowering agents in an HFpEF setting. However, the role of ARNi in delaying or preventing the development of hypertension-mediated organ damage or the progression to HFpEF should be further investigated [62].

Undoubtedly, since the development of ARNI, the use of natriuretic peptides as potential therapeutic agents has grown in importance in the management of HF, particularly for diagnostic, therapeutic, and prognostic use. However, conflicting results were observed with the use of natriuretic peptides in acute HF. Clinical trials and metanalysis raised concerns regarding the use of the recombinant BNP nesiritide in acute HF, particularly for the evidence of conflicting results regarding the study endpoints in different trials and the excessive hypotensive effect [63,64,65].

Similarly, concerns were raised about using the recombinant ANP caperitide and ularitide (a chemically synthesized form of urodilatin) in acute HF. Thus, further studies are warranted to determine the use of natriuretic peptides in acute HF, particularly for the excessive blood pressure-lowering effect [66].

The benefits of SGLT2i have been confirmed across the whole spectrum of LVEF. The EMPEROR-Preserved (Empagliflozin Outcome Trial in Patients with Chronic Heart Failure with Preserved Ejection Fraction) study showed a 21% reduction in the primary composite endpoint of HF hospitalizations and cardiovascular death in patients treated with empagliflozin compared to a placebo independently from gender and LV systolic function [67]. Consistent results have been obtained in the DELIVER (Dapagliflozin Evaluation to Improve the Lives of Patients with Preserved Ejection Fraction Heart Failure) study, which demonstrated an 18% reduction in the primary composite outcome of worsening HF or cardiovascular mortality in patients treated with dapagliflozin [68].

The SOLOIST-WHF (Effect of Sotagliflozin on Cardiovascular Events in Patients with Type 2 Diabetes Post Worsening Heart Failure) study evaluated the effects of sotagliflozin in diabetic patients with recently worsening HF, demonstrating a 33% reduction in HF hospitalizations and cardiovascular death independently from LVEF and renal function [69].

In the EMPULSE (A Study to Test the Effect of Empagliflozin in Patients Who Are in Hospital for Acute Heart Failure) study, which enrolled 530 patients with acute de novo or decompensated HF, empagliflozin reduced the primary outcome, defined as a hierarchical composite of all-cause death, total HF events, time to first HF event, or a ≥5-point change from baseline in the Kansas City Cardiomyopathy Questionnaire (KCCQ) total symptom score, independently from LVEF, diabetes, and onset time of HF [70].

Different evidence has shown that SGLT2i also has BP-lowering actions that favorably affect cardiac afterload, ventricular arterial coupling, cardiac efficiency, LV mass index, and cardiac reverse remodeling [71]. Indeed, SGLT2i induces vasorelaxation and promotes a shift from the sympathetic to the parasympathetic nervous system at the baroreceptor level [56]. In a population of 825 patients with both hypertension and diabetes, empagliflozin reduced SBP and diastolic BP (DBP) reduction compared to a placebo (−3.44 mm Hg and −4.16 mm Hg for SBP and −1.36 mm Hg and −1.72 mm Hg for DBP at the dosages of 10 mg and 25 mg, respectively) [72].

In the SACRA (SGLT2i and Angiotensin Receptor Blocker Combination Therapy in Patients with Diabetes and Uncontrolled Nocturnal Hypertension) study, empagliflozin produced a significant reduction in office SBP (−7.9 mm Hg and −4.2 mm Hg in patients younger and older than 75 years, respectively) and 24 h SBP (−11.0 mm Hg and −8.7 mm Hg in patients younger and older than 75 years, respectively) compared to placebo [73]. Also, dapagliflozin reduced office SBP and 24 h SBP by 4.28 mm Hg and 4.45 mm Hg in a placebo-controlled trial conducted in 311 patients, showing a synergistic BP-lowering effect with calcium channel blockers and beta-blockers [74]. Also, canagliflozin has been demonstrated to significantly reduce SBP (−4.0 mm Hg and −4.8 mm Hg at the dosages of 100 and 300 mg, respectively), 24 h SBP (−3.3 mm Hg and −4.9 mm Hg at the dosages of 100 and 300 mg, respectively), and DBP (−1.9 mm Hg and −2.9 mm Hg at the dosages of 100 and 300 mg, respectively) [75]. Consistently, in a post hoc analysis of the CREDENCE (Canagliflozin and Renal Events in Diabetes with Established Nephropathy Clinical Evaluation) trial, canagliflozin reduced BP by 3.5 mm Hg compared to baseline [76].

An increasing body of evidence has proposed additive and synergistic cardiovascular and nephroprotective effects of the combination of SGLT2i and RAASi, consisting in reductions in systemic oxidative stress, inflammation, BP, renal fibrosis, glomerular injury and proteinuria, more significant vasodilation, and diuretic effects and weight loss [77].

A meta-analysis of 34,551 participants has shown that adding SGLT2i to background RAAS inhibitors produced 12% and 7% reductions in the composite outcome of cardiovascular death and HF hospitalizations and composite kidney outcome and cardiovascular death compared with placebo, respectively [78].

Furthermore, effective nutrition interventions combined with the integration of behavioral support also by using telemedicine approaches can be efficaciously adopted into routine clinical care in hypertensive heart disease and HF. Implementing nutrition strategies that have shown benefits in controlled feeding studies may optimistically impact HF outcomes. Dietary Approaches to Stop Hypertension (DASH) diet interventions may be advantageous in HF patients, as shown in different clinical studies [79,80,81].

The DASH diet demonstrated positive effects in chronic symptomatic HF patients, significantly improving exercise capacity and quality of life scores [82].

However, beyond the controlled feeding studies, the ability of the DASH diet to positively impact HF patients has not been extensively studied, and a consensus for comprehensive dietary guidelines remains elusive in HF patients. In this regard, precision nutrition approaches can foster the understanding and assessment of tailored dietary approaches in HF patients to integrate and improve the clinical care and management of HF patients [83].

### 3.3. New Drugs for BP Management in HF Patients

New drugs have been studied in HF patients exerting a favorable effect on blood pressure and with promising good effects on cardiovascular outcomes. Recent studies have investigated the cardiovascular and renal effects of finerenone, a nonsteroidal selective aldosterone receptor antagonist.

In the FIGARO-DKD (Finerenone in Reducing Cardiovascular Mortality and Morbidity in Diabetic Kidney Disease), which enrolled 7347 patients, finerenone reduced the primary composite endpoint of cardiovascular death, nonfatal myocardial infarction, nonfatal stroke, or hospitalization for HF [84]. Finerenone reduced new-onset HF and improved other HF outcomes in patients with chronic kidney disease and type 2 diabetes, irrespective of a history of HF [85].

The ongoing FINEARTS-HF (Finerenone in Heart Failure Patients) study investigates the efficacy and safety of finerenone in morbidity and mortality in patients with HF and LVEF ≥ 40% [86]. The FIDELITY analysis included 13,026 patients with type 2 diabetes and chronic kidney disease who were randomized to finerenone or placebo. Compared with placebo, finerenone significantly reduced the risk of first hospitalization for HF (−22%), cardiovascular death or first HF hospitalization (−17%), and cardiovascular death or recurrent HF hospitalization (−18%). HF hospitalization was the main driver of the cardiovascular benefit of finerenone in a population that excluded patients with HFrEF at the run-in visit [87].

Finerenone improved HF outcomes irrespective of baseline renal function. Besides the effects on fluid overload, finerenone might also reduce the production of reactive oxygen species and fibrosis and improve endothelial dysfunction and vascular stiffness [88].

These proposed pathophysiological mechanisms have been confirmed in preclinical studies, showing a synergistic effect of the combination of finerenone and empagliflozin in reducing hypertension-mediated organ damage [89].

A large body of evidence has demonstrated the pivotal role of the endothelin-1 (ET-1) system in the development of HFpEF. Indeed, ET-1 receptors are widely expressed in smooth muscle cells, endothelial cells, cardiomyocytes, fibroblasts, macrophages, glomeruli, and tubular cells, mainly mediating vasoconstriction, but also renal water and sodium reabsorption, mitogenic, inflammatory, apoptotic processes, and cardiac hypertrophy [90,91,92]. Overexpression of ET-1 has been linked with several cardiovascular diseases and has a prognostic value in predicting hospitalization and mortality [93].

The ET-1 receptor antagonists bosentan, ambrisentan, and macitentan are currently approved for the treatment of pulmonary hypertension. Different studies have investigated this pharmacological class’s effects in treating hypertension and HF [94]. In a population of 293 hypertensive patients, bosentan (500 or 2000 mg) resulted in a significant reduction in diastolic BP compared to placebo, with a BP-lowering effect equivalent to enalapril. However, the incidence of adverse events, such as headache, flushing, leg edema, and asymptomatic increases in serum aminotransferase levels, was significantly higher in the bosentan group [95]. Similar results have been achieved with the selective type A ET-1 receptor darusentan. In the DORADO trial, which included 379 patients with resistant hypertension who were receiving three or more antihypertensive drugs, atrasentan (50 mg, 100 mg, or 300 mg once daily) resulted in a significant reduction in BP, but with a significantly higher incidence of adverse events, and remarkably, fluid retention [96]. In the DRA-132 study conducted on 849 patients with resistant hypertension, darusentan had neutral effects on sitting office BP but significantly reduced 24-h systolic BP [97].

In animal models of hypertension, aprocitentan, an orally active, dual ET receptor antagonist, resulted in a dose-dependent decrease in BP with synergistic effects with ACEi/ARBs. Moreover, the dual blockade of type A and B receptors seemed to be associated with a lower risk of fluid retention and vascular leakage compared to type A selective blockade, which causes nonselective vasodilation and vasopressin release due to overstimulation of type B receptors [98] In the PRECISION (PaRallEl-group, Phase 3 study with aproCItentan in Subjects with ResIstant HypertensiON) study including 730 patients, aprocitentan significantly reduced unattended, automated office SBP compared to placebo when added to standardized combination background therapy for resistant hypertension over 48 weeks of treatment. The most frequent adverse event was mild-to-moderate edema or fluid retention [99]. Also, a meta-analysis showed that ET-1 receptor antagonists significantly lowered 24-h SBP (−7.6 mm Hg), 24-h DBP (−5.9 mm Hg), office SBP (−6.1 mm Hg), and DBP (−3.8 mm Hg) compared to placebo, independently from diabetes and history of cardiovascular disease [100].

Based on these results, dual ET receptor antagonists might represent a novel antihypertensive therapeutic strategy with potential additive effects when combined with other BP-lowering drugs. However, further studies are needed to evaluate the potential benefits of ET receptor antagonists on organ damage, HF, and cardiovascular outcomes [93].

In HFpEF models, preclinical studies have investigated the impact of the inhibition of type A and B ET receptors on LV structure and diastolic function. In a mouse model of HFpEF, macitentan reduced cardiomyocyte size and aldosterone-induced cardiomyocyte hypertrophy and decreased brain natriuretic peptide mRNA expression [101]. Also, macitentan reduced wall thickness and heart weight in HFpEF and reduced cardiomyocyte size, transcript expression of myocardial brain natriuretic peptide, collagen content, and the titin transcript variant n2b expression. These effects were independent of BP reduction. These data are consistent with previous findings about the action of ET-1 on fibroblast proliferation and the secretion of extracellular matrix proteins [101].

In HFpEF patients, however, the selective inhibition of the ET-A receptor with sitaxsentan improved only exercise tolerance without significant effects on LV structure and impaired diastolic function.

On the other hand, the ENABLE (Endothelin Antagonist with Bosentan and Lowering of Events) [102] and REACH-1 (Research on Endothelin Antagonism in Chronic Heart failure) trials showed that bosentan increased the risk of hospitalizations in patients with chronic HF, probably due to acute fluid retention. Based on the available evidence, ET-1 antagonists do not represent a reliable therapeutic strategy in the management of HF, and their clinical use remains limited. Pulmonary hypertension is due to primary pulmonary vascular disorders [91].

It has been hypothesized that the failure of studies with ET-1 receptor antagonists should be mainly attributed to fluid retention, particularly in patients with HF and renal insufficiency. In addition, vasodilatation might produce a fall in perfusion pressure with consequent activation of RAAS and SNS [103].

The effects of vasopressin receptor antagonists have also been investigated. Indeed, the stimulation of type 1 vasopressin receptor (V1R) induces vascular constriction with increased systemic vascular resistance and afterload and reduced coronary circulation and cardiac contractility. In contrast, the stimulation of type 2 receptor (V2R) is responsible for free water reabsorption and congestion with edema and hyponatremia [104]. However, despite the efficacy of achieving negative water balances, neutral effects on cardiovascular outcomes have been obtained in the different studies performed with vasopressin antagonists [105]. On this basis, European guidelines limit the use of this pharmacological class, particularly tolvaptan, an orally active selective V2R antagonist, to HF patients with persistent hyponatremia and congestion to increase serum sodium and diuresis [13].

Small interfering RNAs (siRNAs) represent a novel promising therapeutic approach. Zilebesiran, the first-in-class antihypertensive siRNA, specifically targets angiotensinogen synthesis in the liver, thus suppressing the downstream RAAS cascade with potential BP-lowering effects [106,107]. Phase I studies have shown a sustained long-term effect, with circulating angiotensinogen reduced by >90% for six months after a single subcutaneous dose (800 mg) and sustained reductions in BP (24 h SBP −15 mm Hg) at eight weeks. In addition, zilebesiran had a favorable tolerability profile, with only mild-to-moderate injection site reactions and no serious adverse events [106,107]. A recently published phase 1 study, which included 107 individuals, showed that dose-dependent decreases in serum angiotensinogen levels and 24-h ambulatory BP were sustained for up to 24 weeks after a single subcutaneous dose of zilebesiran of 200 mg or more [108]. In the phase 2 KARDIA-1 (RNA Interference with Zilebesiran for Mild to Moderate Hypertension) study, which enrolled 377 subjects (302 receiving zilebesiran and 75 receiving placebo) with mild to moderate hypertension, treatment with zilebesiran across a range of doses (150, 300, or 600 mg once every six months or 300 mg once every three months) significantly reduced 24-h mean ambulatory SBP [109]. Preliminary results of the KARDIA-2 (Zilebesiran in Combination with a Standard-of-Care Antihypertensive in Patients With Inadequately Controlled Hypertension) study showed that among patients with uncontrolled hypertension, subcutaneous injection of zilebesiran improved 3-month BP control, especially among those treated with indapamide and amlodipine [110]. The 6-month intervals between the drug administration might represent a tool to improve treatment adherence and achieve effective BP control.

Promising results have also come from studies with selective inhibitors of aldosterone synthase. In a phase 2 study conducted on 248 patients with resistant hypertension, baxdostrat (0.5 mg, 1 mg, or 2 mg) produced significant dose-related reductions in BP compared to placebo [111].

## 4. Conclusions

Hypertension represents one of the most critical risk factors for the development of HF, which is involved in different underlying pathophysiological mechanisms that lead to cardiac hypertrophy, fibrosis, oxidative stress, LV diastolic and systolic dysfunction, and finally, the overt clinical syndrome. Reduction of blood pressure is relevant for HF prevention and HF progression. Several pieces of evidence suggest that tight blood pressure control could be beneficial on HF progression to avoid an increased risk of adverse outcomes in HF patients. Nevertheless, the available data are inconclusive on BP targets in some categories of patients, including old and frail patients. In the last few years, new pharmacological classes with antihypertensive actions have also demonstrated significant efficacy in the treatment of HF, such as SGLT2i and new non-steroidal MRA. On the other hand, other proposed agents, such as ET-1 receptor antagonists, have provided contrasting results. Novel promising strategies could be represented by siRNA and selective aldosterone synthase inhibitors, whose favorable effects have been demonstrated in phase-2 clinical trials.

## Figures and Tables

**Table 1:** Blood pressure targets in patients with and without heart failure.

Population	BP Targets
Hypertensive patients aged <65 years, independently of the presence of HF	<130/80 mm Hg
Hypertensive patients 65 to 79 years old, with or without HF	The primary goal is <140/80 mm Hg, but a target < 130/80 mm Hg can be considered if treatment is well tolerated
Hypertensive patients, with or without HF, independently of age	A target < 120/70 mm Hg should not be actively reached
Hypertensive patients, with or without HF, independently of age	If SBP goals have not been achieved, DBP can be further lowered to <70 mm Hg
Frail patients	Treatment targets should be individualized

**Table 2:** Effects of old and new blood-pressure-lowering drugs effects in heart failure.

Pharmacological Class	Main Effects	Clinical Use
ACE inhibitors	BP reduction, reduction of preload and afterload, improvement of cardiac and vascular remodeling	Hypertension, HFrEF, HFpEF with elevated BP
ARBs	Comparable to ACE inhibitors	Hypertension, HFrEF if ACE inhibitors are not tolerated, HFpEF with elevated BP
ARNi	Reduction of cardiac and vascular remodeling, increase of natriuresis and diuresis, improvement of neurohormonal dysfunction	HFrEF, HFpEF, particularly in the lower EF spectrum, potential use in hypertension
Beta-blockers	Improvement of neurohormonal dysfunction, reduction of cardiac oxygen use and arrhythmias	Hypertension, HFrEF, HFpEF with elevated BP
MRA	Reduction of cardiac fibrosis and remodeling, increased excretion of sodium and water	Resistant hypertension, HFrEF, HFpEF, particularly in the lower EF spectrum
SGLT2i	Cardiac reverse remodeling, vasorelaxation, diuresis	HFrEF, HFpEF, potential use in hypertension
ET-1 receptor antagonists	Pulmonary and systemic vasorelaxation	Pulmonary hypertension, potential use in systemic hypertension and HFpEF
siRNAs targeting angiotensinogen	Inhibition of angiotensinogen synthesis in the liver, thus suppressing the downstream RAAS cascade	Potential use in hypertension and HF prevention

## Data Availability

Not applicable.
